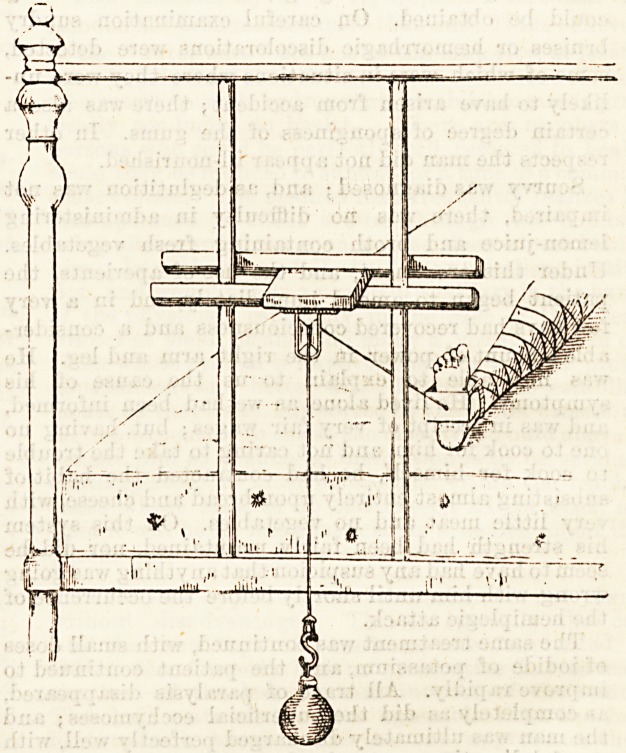# Treatment of Hip-Joint Disease

**Published:** 1893-12-16

**Authors:** 


### PADDINGTON GREEN CHILDREN'S HOSPITAL.

### Treatment of Hip-Joint Disease.

The primary element in the treatment of hip-joint disease is absolute rest. Being a disease of considerable frequency in children, in nearly every case tubercular in character, and often with an onset so gradual and insidious as for some time to escape detection, it is all important that early diagnosis be made, as much may be done by treatment to effect a complete cure, and so escape the various complications which accompany it in its advanced stages. If a child, in the early stage, be kept for some weeks at absolute rest it usually completely recovers. Hence, even though the diagnosis be uncertain by reason of having only one or two slightly marked symptoms, the rest can do the child no harm. If, however, the child is allowed to go about and only "watched" till the symptoms develop, much may have been done to make the case a more or less prolonged one.

In the treatment of out-patients the double Thomas splint is mostly used. This insures absolute rest. It is provided with a pelvic band, in addition to the ordinary shoulder one. This comes off just above the commencement of the gluteal bend, so that the sides of it pass round the pelvis midway between the top of the trochaneter and the iliac crest. This fixes the pelvis thoroughly. The leg pieces are abducted to a slight extent, and usually require a certain amount of outward rotation. The splint is applied next the skin. The child's clothing is all made to fasten up the front in the fashion of a dressing-gown. The various articles of clothing being then laid out flat, and the child in its splint placed on them, they can be fastened round it without producing any tremor or movement which might cause pain. The child is taken out of its splint as seldom as possible. Every morning it is to be sponged all over while in the splint, the various parts of the splint being guarded by a mackintosh or a towel. The general health is always carefully attended to, the child being put upon a course of tonics, and if the state of the digestive organs permits it, on cod liver oil and the syrup of the phosphate of iron. Usually there is no local treatment required.

In many cases, however, owing to the exposure of the double Thomas splint, a single one is used. It has usually to be fitted more or less to each individual case after it comes from the instrument maker, care being taken that it fits accurately and well. It is also provided with a pelvic band. The great drawback to the use of the single splint is that unless the child is carefully watched it is sure to attempt to get on its feet, even when the case is an acute one. To prevent this it is well to have the splint guarded by a "nurse," *i.e.*, a piece of iron projecting from the extremity and continuous with the back piece. This is about one foot in length and has the end curved round on itself. The splint is applied as in the case of the double one; the management of the clothing, &c., being the same. So soon as the acute stage has subsided, the child is allowed to go about on crutches, and with a patten fully four inches in depth. Of course, at this time in both cases the single splint is used. When permanency of cure seems probable, usually in from one to two years, the splint is discarded, then after a time the patten, and finally the crutches.

In severe cases, where there is much pain and crying out at night, and where much flexion, &c., of the hip is present, the child is always treated as an in-patient, at any rate, till the acute symptoms subside. If the onset has been sudden, careful inquiry must be made as to whether it may not be of rheumatic or pyæmic origin, or possibly due to an acute osteo-myelitic condition, which more or less call for their own special line of treatment, in addition to that of absolute rest. This latter is brought about by placing the child in extension. It is placed on a hair mattress, beneath which a fracture-board is laid. Round the child's foot and ankle is wrapped a strip of boracic lint. Two pieces of coarse sticking-plaster are taken, each about two inches broad, and extending from one inch below the sole of the foot to six inches above the knee. These are laid along the leg, one on either side; the plaster side next the skin. They are fixed above the knee by a narrower strip of strapping encircling the leg. The leg is then bandaged from the ankle upwards, and when this band is reached the free ends of the strapping are turned down and fixed by a turn or two of the bandage. A small oblong piece of wood about two and a-half inches across is taken and pierced centrally with a small hole. Attached to its two opposite and narrower sides are pieces of coarse webbing bearing buckles at their free ends. To these are fixed the pieces of strapping projecting beyond the soles of the feet. Through the central hole is passed a stout cord, which glides over a pulley fixed by a special arrangement (see diagram) to the end of the cot, and carrying a hook to which can be suspended a canvas bag, into which a weighed quantity of large shot is placed. The weight required is usually from two to five pounds.

The child is not provided with a pillow, and to prevent it raising itself up in bed it is fixed at the shoulders. A piece of strong webbing is passed across the bed and fixed at each side. Another piece the breadth of the child, and with two cross pieces attached, is taken and placed across the child's chest. The cross pieces, which when fastened from loops, are then passed round the child's shoulders and under the strap stretching across the bed, and then fixed. Further, to prevent lateral movement, and to keep the limbs in position, sand bags are laid along on either side of the body and legs. The foot of the bed is then raised by wooden blocks five to six inches in height placed beneath the legs. Fomentations, &c., locally may be required. So soon as all acute symptoms have passed away the child is placed in a double

Thomas' splint, the after-treatment being as previously described.

Should pus form, the abscess is opened as soon as possible. This is usually done by the anterior method. The skin is shaved, thoroughly cleansed, and well rubbed in with strong mixture, equal parts of 1 in 500 perchloride and 1 in 20 carbolic solutions. A four-inch incision is made, passing downwards from just below the anterior superior spine of the ilium and lying between the tensor and the sartorius. The capsule of the joint is exposed, and is opened by a cut parallel to the neck of the bone. Any carious bone is removed. The cavity is washed clean, swabbed out with 1 in 500 perchloride, and well rubbed in with iodoglycerine or iodoform paste. The wound in the capsule having been closed with interrupted catgut sutures and the superficial one by a continuous silk suture, dressings of double cyanide gauze and salicylic wool are applied. The child is placed with the leg in extension and abducted to about 75 degrees. So soon as the wound has thoroughly healed, and the cicatricial tissue has gained sufficient firmness, the child is placed in a single Thomas' splint and allowed to go about with crutches and a patten. This usually is in about three months, and in a further three to six months these are discarded.

## Figures and Tables

**Figure f1:**